# Supplementary material for: Consistent visuomotor adaptations and generalizations can be achieved through different rotations of robust motor modules
Source: Sci Rep. 2018 Aug 23;8:12657. doi: 10.1038/s41598-018-31174-2 (PMC6107677; doi:10.1038/s41598-018-31174-2)
Supplement: Supplementary file 1 — Supplementary Material [file 41598_2018_31174_MOESM1_ESM.pdf]

Consistent visuomotor adaptations and generalizations can be achieved through different rotations of robust motor modules

Cristiano De Marchis[1], Jacopo Di Somma[1,2], Magdalena Zych[2], Silvia Conforto[1], Giacomo Severini[2]*

[1] Department of Engineering, University of Roma TRE, via Vito Volterra 62, Rome, Italy

[2] School of Electrical & Electronics Engineering, University College Dublin, Belfield, Dublin 4, Ireland

Corresponding Author: Giacomo Severini, School of Electrical & Electronics Engineering, University College Dublin, Belfield, Dublin 4, Ireland, giacomo.severini@ucd.ie

Supporting Information

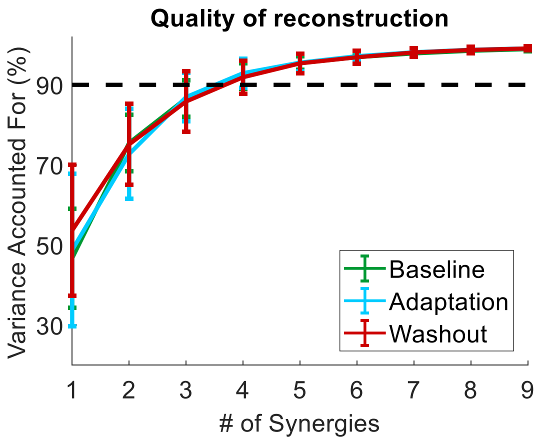

Figure S1. Quality of reconstruction vs. number of synergies extracted. We analyzed the fraction of the total variance (expressed as Variance Accounted For (VAF)) explained by the synergies as a function of the number of synergies extracted. This analysis was performed on the synergies extracted from each subject at baseline (B3), adaptation (Ad3 and Ad6 pooled together) and washout (Wo2 and Wo4 pooled together). Pooling was necessary as adaptation and washout blocks presented reaching trials relative only to 3 targets. By pooling together the last trial of adaptation and generalization (and of the relative washout blocks) we obtain the comparison between the reconstruction obtained from synergies extracted while reaching to 8 targets (B3) and those extracted while reaching to 6 targets (Ad3-Ad6 and Wo2-Wo4). Subjects presented, on average, consistent behaviors during the three phases of the exercise. We found that 4 synergies can satisfactorily reconstruct the original EMG envelopes with an average VAF>90% for all three phases of the exercise.

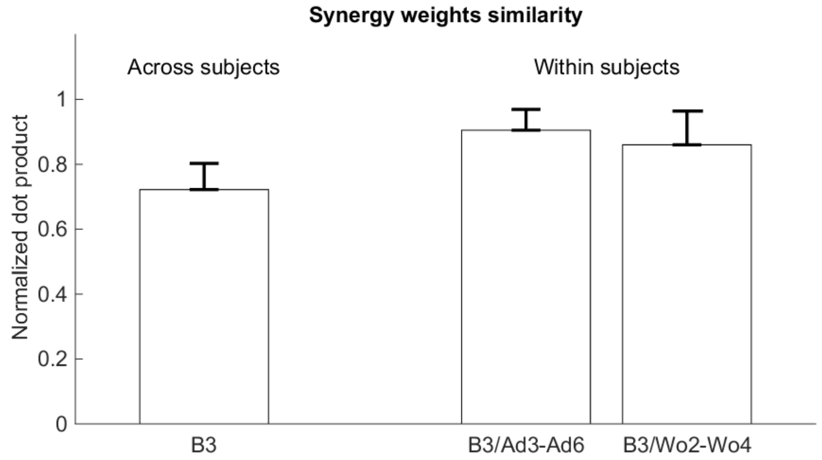

Figure S2. Similarity of synergy weights across and within subjects. Similarity across subjects was calculated, for each subject, as the average normalized dot product between the synergy weights extracted at B3 for the subject and those of the other 13 subjects. Bar plot present the average + standard deviation across subjects. Subject present an average similarity of 0.72±0.08. Similarity between subjects was calculated, for each subject, as the normalized dot product between the synergies extracted at B3 and those extracted pooling together the data for the Ad3 and Ad6 blocks for adaptation and Wo2 and Wo4 for washout. Pooling was necessary as adaptation and washout blocks presented reaching trials relative only to 3 targets. By pooling together the last trial of adaptation and generalization (and of the relative washout blocks) we obtain the comparison between the synergies extracted while reaching to 8 targets (B3) and those extracted while reaching to 6 targets (Ad3-Ad6 and Wo2-Wo4). Bar plots present the average ± standard deviation across all subjects. Subjects presented an average similarity between the synergies extracted at baseline and those extracted at adaptation of 0.91±0.06 and of 0.86±0.10 for those extracted at washout, indicating a remarkable consistency of the synergies across the different phases of the exercise.

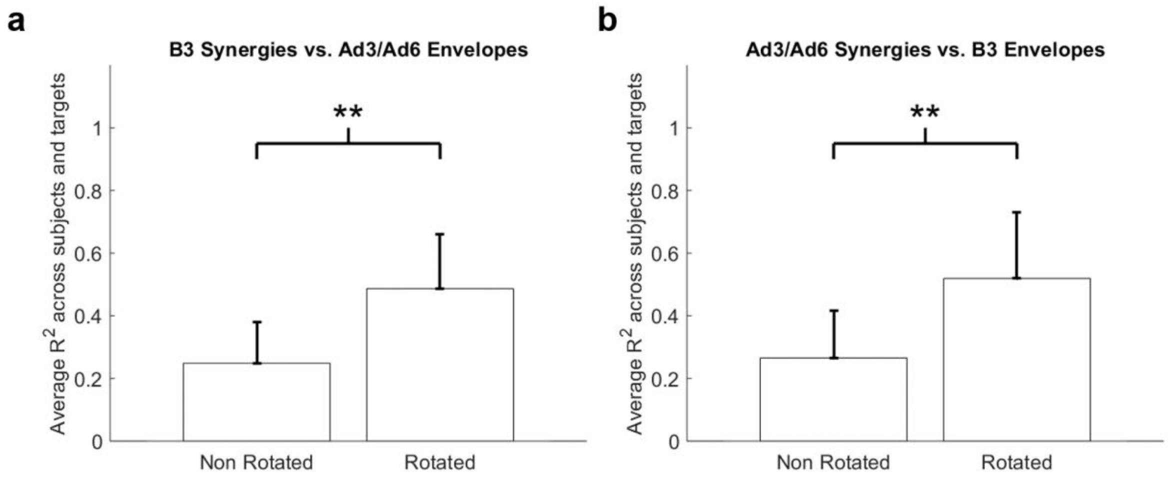

Figure S3. Validation of adaptation as 45° rotation of the synergy APs. The plots present the similarity between the EMG envelopes reconstructed from the synergies analysis and the original ones, for both rotated and non-rotated targets. Panel (**a**) presents the values of similarity between the envelopes reconstructed from the synergies extracted at B3 and the envelopes recorded at Ad3/Ad6, while panel (**b**) presents the values of similarity between the envelopes reconstructed from the synergies extracted at Ad3/Ad6 and the envelopes recorded at B3. Both analyses have been performed on either the original targets ('Non Rotated') or the 45° CCW rotated ones ('Rotated') at B3. For each analysis, 4 synergies were extracted from B3 (**a**) or Ad3/Ad6 (**b**). The average (across movement repetitions) reconstructed (**a**) and original (**b**) envelopes for both the non rotated and rotated B3 targets were calculated and compared with the average (across movement repetitions) original (**a**) or reconstructed (**b**) envelopes at Ad3/Ad6. The plots present the average ± standard deviation across subjects, after averaging across targets. We found that, in both analysis, envelopes from rotated targets (either original or reconstructed after synergies analysis) were more similar to the Ad3/Ad6 envelopes (either original or reconstructed after synergies analysis) in a statistically significant way (based on Wilcoxon's signed rank test with $\alpha = 0.05$, ** denotes significance with $p<0.01$). In panel (**a**) the p-value of the statistical comparison is $p<0.001$ (z-value = -3.296); in panel (**b**) the p-value of the statistical comparison is $p<0.001$ (z-value = -3.296). It is worth noticing that the values of R^2 between the rotated and the original targets, although higher than the ones observed for the non rotated targets, are not as high (0.49 ± 0.17 for panel (**a**), 0.52 ± 0.21 for panel (**b**)) as expected if adaptation was achieved by rotating the synergies by 45°. This result further confirms that adaptation, in our experiments, is not achieved by rotating the synergies by exactly 45° and is consistent with what observed in **Fig. 4**.
